# Thomas Bell

**Published:** 1880-07

**Authors:** 


*Thomas Bell, F. R. S., &c.*—Mr. Thomas Bell, whose death we recorded last week, was the son of a surgeon in large practice for more than fifty years at Poole, in Dorsetshire. He was born on the 11th of October, 1792. He commenced his professional studies with his father. In 1813 he proceeded to London, and attended the practice of Guy's and St. Thomas's Hospitals in the time of Mr. Cline, Sir Astley Cooper, Mr. Travers, and other celebrated surgeons of that period. In 1815 he passed the College of Surgeons, and in 1817 began lecturing at Guy's Hospital on the Anatomy and Diseases of the Teeth. He was appointed at the same time Dental Surgeon to the Hospital.

He also gave lectures on Comparative Anatomy in the same school. Mr. Bell resided for many years in New Bond Street, City, and had a large and select practice as a dentist, and attained a very high position in the scientific world. In 1836 he was appointed Professor of Zoology in Kings College, London, when he ceased his Lectures on Comparative Anatomy at Guy's. He was elected a Fellow of the Royal Society in 1828, and in 1848 was chosen secretary of that body, and held the office till his election as President of the Linnæn Society, in 1853. Mr. Bell became an honorary fellow of the Royal College of Surgeons in 1844, and was formerly one of the Board of Examiners in Dental Surgery at the College. Besides numerous contributions to the Transactions and Proceedings of the Zoological and Linnæn Societies, he was the author of a work on the "Anatomy and Diseases of the Teeth," "History of British Quadrupeds," History of British Reptiles," Gilbert White's "Natural History of Selborne," published in 1876. He gave up practice some years back, and retired to the Wakes at Seeborne, Gilbert White's house, which he had purchased from the family of the naturalist, and where he died on the 13th of March, in the eighty-eighth year of his age.—*The Lancet*

---

